# Supplementary material for: Exogenous Albumin Is Crucial for Pig Sperm to Elicit In Vitro Capacitation Whereas Bicarbonate Only Modulates Its Efficiency
Source: Biology (Basel). 2021 Oct 26;10(11):1105. doi: 10.3390/biology10111105 (PMC8615011; doi:10.3390/biology10111105)
Supplement: Supplementary file 1 [file biology-10-01105-s001.zip › Suppl Table 1.pdf]

Supplementary Table S1. Percentages of viable spermatozoa with an exocytosed acrosome ($PNA^-/EthD\text{-}1^-$) following incubation of boar spermatozoa with different concentrations of bicarbonate (Bic; 0 mM, 5 mM, 15 mM, 38 mM) and BSA (0, 5 mg/mL) for 300 min (IVC). Progesterone (10 µg/mL) was added at 240 min) to induce acrosome exocytosis. Different superscripts (*a-f*) indicate significant differences ($p<0.05$) between treatments (rows) within the same time point. Data are shown as mean ± SEM for 12 independent experiments.

Media	0 min	120 min	240 min	245 min	270 min	300 min
No BSA / No Bic	6.5 ± 0.3^{a}	6.9 ± 0.3^{a}	$7.3 \pm 0.4^{a,b}$	$7.4 \pm 0.4^{a,b}$	$5.9 \pm 0.3^{a,b}$	2.1 ± 0.1^{a}
BSA / No Bic	8.2 ± 0.5^{b}	6.8 ± 0.4^{a}	$12.7 \pm 0.8^{c,d}$	17.9 ± 1.0^{c}	15.1 ± 0.9^{c}	12.2 ± 0.7^{b}
BSA + 5 mM Bic	6.3 ± 0.4^{a}	7.2 ± 0.4^{a}	13.6 ± 0.8^{c}	14.8 ± 0.9^{d}	13.6 ± 0.8^{c}	12.3 ± 0.7^{b}
No BSA + 5 mM Bic	5.9 ± 0.3^{a}	5.4 ± 0.3^{b}	6.3 ± 0.4^{a}	6.8 ± 0.4^{b}	5.1 ± 0.3^{a}	3.4 ± 0.2^{c}
BSA + 15 mM Bic	8.2 ± 0.5^{b}	7.5 ± 0.4^{a}	11.6 ± 0.6^{d}	18.8 ± 1.1^{c}	13.4 ± 0.8^{c}	12.3 ± 0.7^{b}
No BSA + 15 mM Bic	6.2 ± 0.3^{a}	5.5 ± 0.3^{b}	6.8 ± 0.4^{a}	$7.9 \pm 0.4^{a,b}$	7.4 ± 0.4^{d}	3.5 ± 0.2^{c}
BSA + 38 mM Bic	6.5 ± 0.4^{a}	6.6 ± 0.4^{a}	8.2 ± 0.5^{b}	8.6 ± 0.5^{a}	$6.5 \pm 0.4^{b,d}$	3.1 ± 0.2^{c}
No BSA + 38 mM Bic	6.3 ± 0.3^{a}	4.9 ± 0.3^{b}	4.5 ± 0.2^{e}	2.9 ± 0.2^{e}	1.4 ± 0.1^{e}	0.2 ± 0.0^{d}
